# Satisfaction of Clinical Teachers on Standardized Residency Training Program (SRTP) in China: A Cross-Sectional Survey

**DOI:** 10.3390/ijerph19095676

**Published:** 2022-05-06

**Authors:** Boyan Chen, Xiaoyuan Jin, Jie Zhou, Ying Chen, Hongmei Wang

**Affiliations:** 1 Department of Social Medicine of School of Public Health and Department of Pharmacy of the First Affiliated Hospital, Zhejiang University School of Medicine, Hangzhou 310058, China; chen_b_y@zju.edu.cn (B.C.); xiaoyuanj@zju.edu.cn (X.J.); zhou_jie98@zju.edu.cn (J.Z.); chenying1996@zju.edu.cn (Y.C.); 2 Zhejiang Provincial Center for Disease Control and Prevention, Hangzhou 310051, China

**Keywords:** residents, clinical teachers, standardized residency training program (SRTP), cognitive job satisfaction

## Abstract

Background: The Standardized Residency Training Program (SRTP) is a significant initiative to deepen health systems and medical education in developing countries like China. Despite the promotion of the SRTP nationwide and its implementation with various improvements, Chinese continuous medical education is still in its infancy. Compared with the residents, little is known about clinical teachers under the SRTP in China. However, clinical teachers effectively determine the training quality as critical disseminators of knowledge, skills, and values in medical practice. Thus, the study aims to analyze critical factors affecting their cognitive job satisfaction and provide continuous improvements for SRTP. Methods: From 1 December 2018 to 31 May 2019, we conducted a self-designed questionnaire with 13 SRTPs (including both training bases and professional bases) in Shaoxing city to evaluate clinical teachers’ satisfaction. Altogether, 574 clinical teachers responded to the survey expressing generally high overall satisfaction. We adopted a Chi-square test and Fisher’s Exact Test to evaluate the single impact factors affecting the satisfaction of clinical teachers. The multiple factors analysis applied the logistic regression model. Results: The male clinical teachers had significant differences in satisfaction with the teaching content (OR: 0.675, [95% CI: 0.477~0.953]), conflicts between study and work (OR: 0.542, [95%CI: 0.371~0.791]), the attention of leaders (OR: 0.403, [95%CI: 0.252~0.645]), and the subsidies of teachers (OR: 0.527, [95%CI: 0.347~0.805]). Compared with internal medicine, clinical teachers from surgery (OR: 2.396, [95%CI: 1.365–4.206]) and other departments (OR: 2.409, [95%CI: 1.406–4.129]) were more satisfied when they considered that residents have high motivation to attend training. In addition, compared with the attending physicians, the deputy chief physicians (OR: 0.493, [95%CI: 0.310–0.783]) and the chief physicians (OR: 0.683, [95%CI: 0.471–0.991]) disagreed more regarding the residents’ wage being good enough. Conclusion: Clinical teachers widely recognize the SRTP. However, teachers’ satisfaction varied due to different genders, working departments, and professional titles. The study also discussed possible reasons and strategy implications behind these findings, which combined unique Chinese society characteristics. Further, we believe the analysis and interpretations remind us of the applications of residency training methods from other Western countries, which should also consider the unique socio-cultural challenges.

## 1. Introduction

The international medical community recognizes that residency training is crucial for building a high-quality medical team, improving the overall medical services level, and ensuring the quality of care [1,2]. In Europe and the United States, residency training has been standardized and formed as a relatively complete management system following hundreds of years of development; there are even readymade complete training systems to train medical doctors at all levels, including entry-level, postgraduate, and continuing medical education [3,4,5,6]. Drawing from world experience, China has also focused on developing residency training programs in the past few years.

### 1.1. Standardized Residency Training Program (SRTP)

Since 2014, the National Health and Family Planning Commission (NHFPC) in China has implemented the Standardized Residency Training Program (SRTP) which requires all graduate medical students to complete a 3-year training before starting their careers. [7,8,9] Differing from the American type of residency program, which was launched in some university-affiliated hospitals, the SRTP was implemented nationwide in both training bases (medical and health institutions, e.g., hospitals) and professional bases (professional clinical departments) [7,10]. Specifically, the training bases chose to be sufficiently professional—receiving no criticism of medical safety incidents in the past three years—and to train residents’ basic clinical practices by rotating the significant departments of two-level disciplines. The professional bases have rigorous requirements for faculty conditions (e.g., *a bed utilization rate of ≥85%; an annual number of inpatient admissions ≥ 3800; and an annual outpatient volume ≥ 100,000*) to train residents’ clinical skills in a particular department of three-level disciplines. [7,8,10,11,12].

Until 2020, the promotion of the SRTP increased the cumulated number of residents nationwide to 332,000 (recoded as 480,000 if including the postgraduate specialties); 45,000 residents trained as general practitioners and 20,000 residents worked in pediatrics after completing the training [9]. Further, as a significant initiative to deepen health systems and medical education [1], the SRTP also trained qualified clinicians resulting in various improvements, from improving teaching quality, providing comfortable study settings, and enlarging clinical cases as teaching contents. According to the reports from the National Medical Examination Center (NMEC), the pass rates of residents who participated in the SRTP for the first time in the clinical practice qualification examination in 2018 were 20% higher than those who did not receive some training from the SRTP [13]. From 2017 to 2018, 120,000 residents successfully passed the complete examination and obtained training certificates [13].

### 1.2. Clinical Teachers

Clinical teaching is the cornerstone of medical students’ professional development; thus, clinical teachers effectively determine the training quality as critical disseminators of knowledge, skills, and values in medical practice. The SRTP involves clinical teachers with rigorous certification—only a physician with at least three years of experience as an attending physician, or above, can work as a clinical teacher [10]. The faculty training requires teachers to receive uniform qualifications and be familiar with the standardized training regulations [10,14,15]. Further, the SRTP limits the number of supervised residents per each clinical teacher, who cannot train more than three simultaneously [9,10].

Despite the promotion of the SRTP nationwide and its implementation with various improvements, Chinese continuous medical education is still in its infancy. In its developed stage, the training to become a resident doctor depends on the hospital employed, leading to differential medical levels across regions. Moreover, the imbalance and inadequacy in its development have conflicted with increasing medical demand [16]. Thus, the SRTP has increasingly attracted more attention from all walks of life, primarily in academic studies. In the previous studies, many researchers sought to understand residents’ perceptions about the SRTP and observe their mental health [16,17,18,19]. Moreover, many studies adopt a mature approach, testing residents’ satisfaction and collecting their evaluations regarding training organizations [16,20,21]; they report that training from the SRTP is beneficial. Approximately one-third of learning in the clinical setting originates from clinical teachers [1].

However, compared with our knowledge of residents, little is known about clinical teachers under the SRTP in China. The limited studies discussed that clinical teachers have close relationships with their resident students to provide optimal teaching approaches, contribute much to the residents’ studies, and even spend up to one-fourth of their time supervising, instructing, and evaluating students [1,3]. The positive teaching approach and perceived high-quality education for residents are undoubtedly associated with high overall satisfaction with the SRTP [20]. Herein, this study aimed to investigate the status quo and the factors affecting clinical teachers’ cognitive job satisfaction and provide proper advice and measures for the sustainable development of standardized training. Moreover, we believe in the importance of the perspective of clinical teachers; analyzing their satisfaction with the SRTP is beneficial for recommending strategies for national resident training.

## 2. Materials and Methods

### 2.1. Study and Population Design 

The study applied the simple random sampling method to select 13 residency standardization bases in Shaoxing, Zhejiang Province. We adopted stratified random sampling from the multi-bases to recruit 600 clinical teachers as a representative sample. We developed an anonymous survey in the Chinese language to evaluate cognitive job satisfaction, from 1 December 2018 to 31 May 2019. To minimize the sampling bias, we set the inclusion criterion of teachers who had teaching experience of more than one year. The recruitment provided a brief synopsis of the research aims and design and enrolled clinical teachers who were available, willing, and interested. In addition, the study asked the recruited teachers to scan the “Quick Response (QR) Code” and enter the WeChat Mini Program (*Wenjuanxing*) to answer the questionnaire independently within the specified time. The researchers provided in-person technical assistance on barriers to using the online platform to control data quality. After receiving participants’ submissions, the valid data involved in the study analysis (*n* = 574, 95.7%) were reviewed by the researchers.

### 2.2. Questionnaire Design

The questionnaire was self-designed by carrying out relevant policy documents reviews, literature reviews, and consultations with experts. The final distributed version of the survey was formed from the repeated revisions of the pre-investigation. The self-administered questionnaire had 19 items and included three sections: (1) demographic information, (2) career information, and (3) perceived cognitive job satisfaction.

#### 2.2.1. Cognitive Job Satisfaction

Cognitive job satisfaction aimed to evaluate participants’ cognitions about the job. [22] The study divided satisfaction into positive attitude (PA) and negative attitude (NA), including ten items. In particular, the questionnaire design applied the Job Descriptive Index (JDI) [23], which asks participants to rate their satisfaction with specific facets of their job as the foundation. Previous studies about residents’ satisfaction in domains including training policy, the management system, and the training returns with the SRTP were also reviewed [24,25,26]. We adapted a new measure with ten items which were evaluated at item level in our study. The answers from participants were collected into “yes” and “no” for PA and NA, respectively. When evaluating teachers’ satisfaction with the teaching subsidies and training base supplements, the original response options were “very satisfied,” “satisfied,” “a little satisfied,” “dissatisfied,” and “very dissatisfied.” The five options were divided into “PA” (“very satisfied,” “satisfied,” and “a little satisfied”) and “NA” (“dissatisfied” and “very dissatisfied”).

#### 2.2.2. Individual Covariates

Section 1 consisted of necessary socio-demographic information: gender, age, marital status (recoded into married and other (including unmarried, divorced, and widowed)), and education level (junior college or college, and postgraduate or above). The covariates about career information in Section 2 included working experience (10-year categories), professional titles (attending physician, deputy chief physician, and chief physician), hospital types (TCM hospital, specialized hospital, and general hospital), hospital levels (tertiary hospital, and non-tertiary hospital), and clinical departments (department of internal medicine, surgical department, and others).

### 2.3. Statistical Analyses

The study calculated the frequency, percentage, mean, and standard deviation (SD) to describe the demographic and career information. The study tested univariate analysis by first applying the Chi-square test and Fisher’s Exact Test. Significant variables (*p*-value of ≤0.05) from the univariate analysis were subjected to multivariate analysis. Moreover, the study employed logistic regression analysis to explore the association between the covariates and satisfaction. All statistical analyses were performed using SPSS 18.0 software(SPSS Inc., Chicago, IL, USA), and a variable with a *p*-value ≤ 0.05 was considered statistically significant. The adjusted odds ratios (ORs) and their 95% confidence intervals (CIs) of the independent variables were estimated.

### 2.4. Ethics Statement

The Zhejiang Medical Ethics Committee approved the study as an institutional ethics exemption (https://lunli.wsjkw.zj.gov.cn/ (accessed on 30 April 2020), which involves using educational tests (cognitive, diagnostic, aptitude, or achievement tests) [27]. To minimize the study’s risks and to protect subjects’ identities, the researchers provided informed content to all the recruited participants and presented a brief description of the study before answering the questionnaires, including the purpose, time commitment, and any risks. All the researchers completed the required online training module through the Collaborative Institutional Training Initiative (CITI) Program [28].

## 3. Results

### 3.1. Demographic and Career Information

As shown in Table 1, the study had 530 (92.3%) clinical teachers were under 50 years old. Around half of the participants were female (50.2%), and 97.6% of the instructors were married or cohabited. Of the 574 participants, 76% of the clinical teachers graduated from junior college or college. Four-fifths (79.1%) of the participants had been teaching for 1~10 years. In addition, two-thirds (66.4%) of the participants worked in general hospitals, and 228 (39.7%) clinical teachers worked in tertiary hospitals. More than half (59.8%) of the teachers had deputy chief physician or chief physician titles. The participants were recruited from different hospital departments—67 (11.7%) teachers from the internal medicine department, 204 (35.5%) teachers from the surgical department, and 52.8% from other departments. 

### 3.2. The Satisfaction of Clinical Teachers toward SRTP

The study evaluated the clinical teachers’ satisfaction according to the PA and NA prevalence from ten items (Table 2). Many of the items received a higher percentage of PA. Specifically, 86.9% of the clinical teachers responded positively to the program design, and 86.4% thought the training and promotion design was reasonable. Almost all teachers (96.3%) believed the provided content met teaching requirements, and 94.6% evaluated the SRTP as helpful for students. In addition, 83.1% of the teachers thought administrators had paid enough attention to the training, and 60.8% of clinical teachers thought students were enthusiastic about studying in the SRTP. Moreover, 80.5% of teachers were satisfied with their subsidies. However, more than half of the clinical teachers (54.7%) believed that students’ wages were insufficient. Around half of the participants (47.4%) felt the training period was too long. Nearly one-third (39.2%) of the teachers thought students had conflicts between study and work.

### 3.3. Factors Affecting the Teacher’s Cognitive Job Satisfaction

As shown in Table 3, gender, marital status, and educational level significantly affected clinical teachers’ satisfaction. Significant differences existed between male and female clinical teachers’ satisfaction regarding teaching subsidies (*p* = 0.003), and when evaluating the items “*There is no conflict between studies and work*” (*p* = 0.006) and “*Leaders of medical institutions attach great importance to the SRTP*” (*p* < 0.001). According to the item “*The SRTP does not cause conflicts between residents’ studies and work*”, clinical teachers who were married or cohabited reported higher agreement (*p* = 0.044), and teachers who graduated from junior college or college also reported a higher agreement (*p* = 0.003). 

Furthermore, titles, hospital levels, and working department types were significantly associated with teachers’ satisfaction. Specifically, titles affected teachers’ satisfaction with students’ wages (*p* = 0.008). Among different hospital levels, the significant differences in teachers’ recognition were calculated from 4 items: “*The SRTP does not cause conflicts between residents’ studies and work*” (*p* = 0.038); “*Leaders of medical institutions attach great importance to the SRTP*” (*p* = 0.009); “*The required training period for residents is not too long in the SRTP*” (*p* = 0.017); and “*The residents’ wage is good enough provided by the SRTP*” (*p* = 0.035). There were significant differences among teachers in different hospital types regarding whether training was too long (*p* = 0.020), whether there was no problem with the training and promotion design (*p* = 0.005), whether there was a conflict between study and work (*p* = 0.025), and whether students’ payments were good enough (*p* = 0.035). Moreover, different working departments influenced teachers’ satisfaction with whether there were conflicts between study and work (*p* = 0.046) and whether students were highly motivated (*p* = 0.003). 

Univariate analysis of all variables was detailed in Supplementary File S1.

### 3.4. Logistic Regression Analysis of Teachers’ Cognitive Job Satisfaction

In univariate analysis, variables with statistical significance to clinical teachers’ satisfaction among demographic and career variables were obtained and applied in the logistic regression model. The logistic regression analysis showed that the variables of gender, marital status, types of hospital, title, and working departments influenced clinical teachers’ satisfaction significantly.

Notably, male (OR: 0.542, [95%CI: 0.371–0.791]), graduated as postgraduate or above (OR: 0.612, [95%CI: 0.404–0.926]), unmarried, divorced, and widowed (OR: 0.280, [95%CI:0.093–0.849]), and TCM hospitals’ teachers (OR: 0.466, [95%CI: 0.294–0.739]) were more dissatisfied with “*The SRTP does not cause conflicts between residents’ studies and work*”. Male teachers (OR: 0.403, [95%CI: 0.252–0.645]) reported lower agreement with “*Leaders of medical institutions attach great importance to the SRTP.*” In addition, male clinical teachers (OR: 0.527, [95%CI: 0.347–0.805]) were less satisfied with teachers’ subsidies. Compared with the department of internal medicine, clinical teachers from the department of surgery (OR: 2.396, [95%CI: 1.365–4.206]) and other departments (OR: 2.409, [95%CI: 1.406–4.129]) were more satisfied when they considered “*The residents have high motivations to attend daily training.*” Further, compared with attending physicians, deputy chief physicians (OR: 0.493, [95%CI: 0.310–0.783]) and chief physicians (OR: 0.683, [95%CI: 0.471–0.991]) disagreed more that the students’ wage is good enough (Shown in Table 4).

## 4. Discussion

Most clinical teachers hold a positive attitude and have widely accepted the SRTP based on their evaluation of each item. However, teachers with different personality traits present different opinions after exploring the items in detail. The main findings indicated that most teachers with different genders, working departments, and professional titles have significant differences in satisfaction levels. Although only evaluating the clinical teachers’ overall satisfaction is an inaccurate method to identify issues with the SRTP [29], the possible influential factors and their causes combined with Chinese society characteristics might provide valued recommendations to develop the SRTP in the future.

Initially, the results reported that the male clinical teachers’ evaluations were low because of the unsatisfactory teaching content and the conflicts between students’ study and work. Moreover, compared with their female counterparts, more male teachers considered that the SRTP receives little attention from the leaders, and the provided subsidies to teachers are not good. We propose the Chinese culture’s gendered social role expectations as a cause [20,30]. Notably, cultural traditions and religious convictions have shaped men’s role; in society they are expected to be the chief breadwinner and responsible for supporting their families [31,32]. In contrast, the traditional community assumes women’s role is to do household work such as child-rearing and day-to-day life chores. Although some evidence has stated that promoting gender equality in the workplace has already improved women’s status, the traditional gender roles still appear pervasively. Chinese female employees experienced more conflicts in their roles at home and in the workplace. Because of the contradictory expectations of gender roles, men and women evaluated job satisfaction with different factors [30]. We believed that it was the primary explanation for the lower satisfaction of the male. According to the previous findings, males considered the main determinants include income, responsibility, and professional development opportunities. In contrast, women considered job stability, the balance between work and family, and professional status more critically. In addition, some studies reported that Chinese men attached higher importance to challenging work and valued professional development opportunities more than their female counterparts [33]. Recent research examined the incentives to help improve clinical teachers’ motivation; it found that educators were highly motivated when they felt their leaders value the work of teaching [34]. Therefore, the study recommended that the SRTP manage and spread diverse work types with a different gender. Moreover, the management in the SRTP can develop a commitment to the profession, and alliance and support among colleagues help build close relationships with others.

The results also indicated that most clinical teachers believe that the residents’ enthusiasm for studying in the SRTP is high. However, compared with surgery and other departments’ clinical teachers, the clinical teachers who worked as internal medicine physicians were less satisfied when they considered “*The residents have high motivations to attend daily training*”. Almost all internal medicine departments work with geriatric chronic diseases and severe and complicated cases in China. The current situation leads typical patients with milder conditions to be few, which causes clinical teachers to apply related diseases that cover differential diagnoses and treatments as teaching cases [35,36]. However, the training period of the above studies is limited for residents. During the entire 33 months, residents must attend 29 months of rotation training in the various subspecialties (Cardiology, Nephrology, Gastroenterology, etc.), and four months of rotation training in the elective subjects under the internal medicine department [7,10,37]. In addition to participating in outpatient and emergency work and various teaching activities (teaching visits, case discussions, professional lectures, etc.), residents also have to complete the required number of diseases and basic skills. In short, excessive concentration of learning during the short-term rotation period burdens residents, creating pressure and making their residency training more challenging. 

Communication between residents and patients during outpatient training may be another reason. Many internal medicine patients have long-term illnesses and repeated hospitalization [37,38]. Thus, their minds and emotions are the most depressed. A previous study stated that many patients resent residents; they are reluctant to accept residents’ consultation, body investigation and diagnosis, and treatment operations. This might be a barrier affecting the residents’ study to a certain extent [38]. Therefore, the study recommended that the SRTP strengthen communication with patients to obtain their cooperation and chooses typical cases that are relatively mild and easy to communicate. Specifically, all clinical teachers can select patients to be involved in clinical practice teaching. The educators can communicate with the patients before the class to explain the time, purpose, and time of teaching, obtaining the patients’ and their families’ understanding.

Finally, the deputy chief physician and chief physician disagreed that students’ wages were good enough compared with the attending physician. As a mid-level title, attending physicians are the main force of clinical care in Chinese hospitals. However, most who work in the inpatient setting remain at this stage for the better part of their careers due to the vice chief physician requirements [39]. Under the system with a high degree of competitiveness, the contribution of work to develop the SRTP is an opportunity for attending physicians. Many SRTP training bases have already applied rewarding and punishing policies to evaluate teachers’ performance, including the number of teaching residents and the pass rate of the complete assessment. Most importantly, these results are often related to the reputation of the teachers, regarding issues such as promotion, salary increase, evaluation, and dismissal [37,38,39,40,41,42]. Therefore, the final purpose of providing training in the SRTP for attending physicians is to earn rewards to be promoted as deputy chief physicians. 

For many deputy and chief physicians, promotion no longer has an incentive effect. Instead, broadening the academic field, mastering cutting-edge theories and methods, and seeking medical talents have become their potential needs. Moreover, many hospitals with the SRTP maintain the appropriate proportion of chief physicians to deputy chief physicians to attending physicians to residents, at the ratio of 1:2:4:8 [39]. Under this distribution, except for supervising interns and residents as instructors in the SRTP, the deputy chief and chief physicians are also needed to act as administrative leaders in clinical departments. They are more concerned about residents’ situations besides their income. The study recommended that leaders incorporate resident physicians’ performance appraisal as a reference basis for salary distribution and give resident physicians certain material rewards with excellent daily routines [43,44].

### Study Limitations 

Several limitations of this study occurred and should be addressed in the future. Firstly, although this study adopted multiple random sampling methods, the recruitment of clinical teachers only selected several bases in a single city as the research sample and a large-scale investigation was not conducted due to the limitations on financial support and time. Thus, the future study will add more areas to verify the current findings in other training bases, with different classes and types from various regions. Furthermore, another limitation is the self-designed questionnaire applied in this study. Although existing studies and the adopted satisfaction measures were mature in their application and were validated, the applied subjects forced on the residents and the measured content concerning employee job satisfaction differed from the study objectives. In short, the main findings summarized from this study only serve as the first exploration. The verification of the main findings should be tested in a future study on instrument development and the current status of job satisfaction regarding the SRTP among clinical teachers. Furthermore, future research might consider more contextual factors and curriculum planning in different clinical settings [35,36,39,45].

## 5. Conclusions

The SRTP is a long-term talent development strategy that relies on multi-departmental collaboration. The practical implementation of the SRTP in the future requires the active participation of residents and high-quality training efforts from clinical teachers. However, it also needs the regulation, design, and development of managers and policymakers as another firm support. The main findings from the present research—that gender difference, working departments, and professional titles induced different satisfaction among clinical teachers—provided broad implications and foundations for the future development of the SRTP at the management level. Specifically, we suggested building commitment to the profession, and alliance and support among colleagues, enforcing education about communications between residents and patients, and providing more welfare to solve the issue of low payments to residents.

## Figures and Tables

**Table 1:** Demographic and Career-Related Characteristics of Participants (*n* = 574).

	*n*	%
Sex		
Male	286	49.8
Female	288	50.2
Marital status		
Married or cohabited	560	97.6
Other (i.e., unmarried, divorced, and widowed)	14	2.4
Age (years)		
≤40	297	51.7
41~50	233	40.6
51~60	44	7.7
Education		
Junior college or college	437	76.3
Postgraduate or above	137	23.9
Title		
Attending physician	231	40.2
Deputy chief physician	225	39.2
Chief physician	118	20.6
Hospital level		
Tertiary class	228	39.7
Non-tertiary class	346	60.3
Types of hospital		
TCM hospital	112	19.5
Specialized hospital	81	14.1
General hospital	381	66.4
Teaching experience (years)		
1–10	454	79.1
11–20	96	16.7
21–30	22	3.8
Department		
Internal medicine	67	11.7
Surgery	204	35.5
Others	303	52.8

**Table 2:** The satisfaction of clinical teachers towards SRTP.

Items	Cognitive Job Satisfaction ^a^	
	PA (%)	NA (%)
The SRTP provides enough supported content (e.g., number of diseases and cases) to meet teaching requirements.	553 (96.3)	21 (3.7)
The newly developed design of the SRTP is reasonable.	496 (86.4)	78 (13.6)
There is no problem with personnel policy by the SRTP.	499 (86.9)	75 (13.1)
The SRTP does not cause conflicts between residents’ studies and work.	394 (68.6)	180 (31.4)
Leaders of medical institutions attach great importance to the SRTP.	477 (83.1)	97 (16.9)
The SRTP helps improve residents’ abilities.	543 (94.6)	31 (5.4)
The required training period for residents is not too long in the SRTP.	302 (52.6)	272 (47.4)
The residents have high motivation to attend daily training.	349 (60.8)	225 (39.2)
The residents’ wage is good enough provided by the SRTP.	260 (45.3)	314 (54.7)
Clinical teachers’ subsidies supported by the SRTP are good.	462 (80.5)	112 (19.5)

Note. ^a^ Cognitive job satisfaction: job satisfaction measured by cognitions (evaluative).

**Table 3:** Univariate analysis of demographic and career variables of clinical teachers’ satisfaction (*n* = 574).

Items	Sex ^c^	Marital Status ^c^	Age	Education Level ^c^	Title ^c^	Hospital Level ^c^	Types of Hospital ^c^	Teaching Experiences	Department ^c^
				*p*-Value (*χ*^2^) ^a^					
The SRTP provides enough supported content (e.g., number of diseases and cases) to meet teaching requirements.	0.811 (0.057)	1.000 ^b^	0.619 (0.958)	0.995 (<0.001)	0.967 (0.068)	0.227 (1.459)	0.308 (1.936)	0.394 (1.862)	0.067 (5.411)
The newly developed design of the SRTP is reasonable.	0.135 (2.234)	0.109 ^b^	0.121 (4.231)	0.213 (0.644)	0.107 (4.463)	0.212 (1.560)	0.160 (3.670)	0.441 (1.639)	0.149 (3.802)
There is no problem with personnel policy by the SRTP. ^d^	0.101 (2.697)	0.234 ^b^	0.846 (0.334)	0.368 (0.811)	0.100 (4.602)	0.038 * (4.317)	0.005 ** (10.571)	0.980 (0.041)	0.573 (1.115)
The SRTP does not cause conflicts between residents’ studies and work. ^d^	0.006 ** (7.592)	0.044 * ^b^	0.981 (0.039)	0.003 ** (8.778)	0.135 (4.012)	0.408 (0.685)	0.025 * (7.392)	0.421 (1.730)	0.046 * (6.177)
Leaders of medical institutions attach great importance to the SRTP. ^d^	<0.001 *** (12.183)	1.000 ^b^	0.304 (2.385)	0.763 (0.091)	0.117 (4.296)	0.009 ** (6.817)	0.017 * (8.139)	0.120 (4.245)	0.131 (4.070)
The SRTP helps improve residents’ abilities.	0.869 (0.027)	0.545 ^b^	0.249 (2.777)	0.863 (0.030)	0.245 (2.817)	0.383 (0.762)	0.848 (0.330)	0.670 (0.800)	0.933 (0.138)
The required training period for residents is not too long in the SRTP. ^d^	0.360 (0.838)	0.459 (0.548)	0.099 (4.624)	0.246 (1.348)	0.148 (3.819)	0.017 * (5.686)	0.019 * (7.875)	0.547 (1.208)	0.562 (1.152)
**The residents have high motivation to attend daily training.** ^d^	0.239 (1.389)	0.787 (0.073)	0.545 (1.215)	0.143 (2.143)	0.063 (5.523)	0.093 (2.830)	0.420 (1.737)	0.144 (3.883)	0.003 ** (11.503)
The residents’ wage is good enough provided by the SRTP. ^d^	0.206 (1.602)	0.720 (0.128)	0.181 (3.414)	0.425 (0.636)	0.008 ** (9.596)	0.035 * (4.425)	0.035 * (6.711)	0.737 (0.611)	0.220 (3.024)
**Clinical teachers’ subsidies supported by the SRTP are good.** ^d^	0.003 ** (8.941)	0.742 ^b^	0.295 (2.442)	0.669 (0.183)	0.691 (0.738)	0.745 (0.106)	0.643 (0.884)	0.698 (0.718)	0.076 (5.163)

Note. Measured items which only have one statistically significant associated factor are indicated in bold. ^a^ Values were calculated from the Chi-square test to examine the effects of demographical information and career characteristics on the clinical teachers’ satisfaction (significant at * *p* ≤ 0.05; ** *p* ≤ 0.01; *** *p* ≤ 0.001). ^b^ Values were calculated from Fisher’s Exact Test to examine the effects of demographical information and career characteristics on the clinical teachers’ satisfaction (significant at * *p* ≤ 0.05; ** *p* ≤ 0.01; *** *p* ≤ 0.001). ^c^ Significant variables were calculated into the model with subsequent multivariable analyses. ^d^ Questionnaire items, which have significant factors from univariate analysis, can build logistical models.

**Table 4:** Logistic regression analysis of teachers’ satisfaction.

Model	Characteristic		OR (95% CI)	*p*-Value ^1^
There is no problem with personnel policy by the SRTP.	Hospital level	Tertiary class	Reference	
		Non-tertiary class	1.392 (0.548–3.539)	0.487
	Types of hospital	General hospital	Reference	
		TCM hospital	0.575 (0.218–1.522)	0.256
		Specialized hospital	1.512 (0.486–4.704)	0.476
The SRTP does not cause conflicts between residents’ studies and work.	Gender	Female	Reference	
		Male	0.568 (0.391–0.826)	**0.003**
	Marital status	Recorded married or cohabited	Reference	
		Other (i.e., unmarried, divorced, and widowed)	0.276 (0.091–0.837)	**0.023**
	Education	Junior college or college	Reference	
		Postgraduate or above	0.612 (0.404–0.926)	**0.020**
	Types of hospital	General hospital	Reference	
		TCM hospital	0.514 (0.327–0.807)	**0.004**
		Specialized hospital	1.053 (0.596–1.863)	0.858
	Department	Internal medicine	Reference	
		Surgery	1.482 (0.823–2.668)	0.190
		Others	1.692 (0.962–2.975)	0.068
Leaders of medical institutions attach great importance to the SRTP.	Gender	Female	Reference	
		Male	0.403 (0.252–0.645)	**<0.001**
	Hospital level	Tertiary class	Reference	
		Non-tertiary class	1.828 (0.810–4.126)	0.147
	Types of hospital	General hospital	Reference	
		TCM hospital	0.700 (0.294–1.668)	0.421
		Specialized hospital	1.538 (0.586–4.038)	0.382
The required training period for residents is not too long in the SRTP.	Hospital level	Tertiary class	Reference	
		Non-tertiary class	1.024 (0.536–1.958)	0.943
	Types of hospital	General hospital	Reference	
		TCM hospital	0.585 (0.287–1.193)	0.140
		Specialized hospital	0.689 (0.326–1.459)	0.331
The residents have high motivation to attend daily training.	Department	Internal medicine	Reference	
		Surgery	2.396 (1.365–4.206)	**0.002**
		Others	2.409 (1.406–4.129)	**0.001**
The residents’ wage is good enough provided by the SRTP.	Title	Attending physician	Reference	
		Deputy chief physician	0.493 (0.310–0.783)	**0.003**
		Chief physician	0.683 (0.471–0.991)	**0.045**
	Hospital level	Tertiary class	Reference	
		Non-tertiary class	0.939 (0.490–1.799)	0.850
	Types of hospital	General hospital	Reference	
		TCM hospital	0.542 (0.263–1.115)	0.096
		Specialized hospital	0.687 (0.322–1.470)	0.334
Clinical teachers’ subsidies supported by the SRTP are good.	Gender	Female	Reference	
		Male	0.527 (0.347–0.805)	**0.003**

^1^ Statistically significant associated factors are indicated in bold.

## Data Availability

The online survey collected all used data using the Wenjuanxing Platform (https://www.wjx.cn/app/survey.aspx (accessed on 3 June 2019). More detailed data used and analyzed during the current study are available from the corresponding author on reasonable request.

## Supplementary Materials

The following supporting information can be downloaded at: https://www.mdpi.com/article/10.3390/ijerph19095676/s1, Supplementary File S1: Univariate analysis include all variables (complected version).

## Abbreviations

**SRTP**	Standardized Residency Training Program
**NHFPC**	National Health and Family Planning Commission
**NMEC**	National Medical Examination Center
**PA**	Positive attitude
**NA**	Negative attitude
**CITI**	Collaborative Institutional Training Initiative Program

## References

[1] Seely A.J., Pelletier M.P., Snell L.S., Trudel J.L. (1999). Do surgical residents rated as better teachers perform better on in-training examinations?. Am. J. Surg..

[2] Lian S., Xia Y., Zhang J., Han X., Chi C., Fetters M.D. (2019). Comparison of general practice residents’ attitudes and perceptions about training in two programmes in China: A mixed methods survey. Fam. Med. Community Health.

[3] Bulte C., Betts A., Garner K., Durning S. (2007). Student teaching: Views of student near-peer teachers and learners. Med. Teach..

[4] Cueto J., Burch V.C., Adnan N.A., Afolabi B.B., Ismail Z., Jafri W., Olapade-Olaopa E.O., Otieno-Nyunya B., Supe A., Togoo A. (2006). Accreditation of undergraduate medical training programs: Practices in nine developing countries as compared with the United States. Educ. Health.

[5] Wang Y.Y., Wang R.Y., Zhang J.J., Zhang Y.L., Wang Y.Y., Sun Y.J., Wang J.Y. (2018). Satisfaction of Trainees with the Standardized General Practitioner Training Delivered by the General Practice Department of a General Hospital. Chin. Gen. Pract..

[6] De Andrade F., Griebenow R., Costello R.W., Guenova M., Schaefer R., Chalmers J.D., Tichelli A., Raguz D., Stein J. (2018). The future of accreditation of continuing medical education (CME)-continuing professional development (CPD) in Europe: Harmonisation through dialogue and consensus. J. Eur. CME.

[7] National Health and Family Planning Commission (2014). Guidance of the National Health and Family Planning Commission and Seven other Departments on the Establishment of a Standardized Residency Training System. http://www.nhfpc.gov.cn/qjjys/s3593/201401/032c8cdf2eb64a369cca4f9b76e8b059.shtml.

[8] National Health and Family Planning of the People’s Republic of China (2015). A Report on the Development of Standardized Residency Training in China. http://www.nhfpc.gov.cn/qjjys/s3594/201505/953d3206bb1c4c869944e0a139328a0d.shtml.

[9] National Health Commission of the People’s Republic of China Letter of Response to Proposal No. 0868 (Medical Sports No. 089) of the Second Session of the 13th National Committee of the Chinese People’s Political Consultative Conference. http://www.nhc.gov.cn/wjw/tia/202009/4ea9a46a93374e23916eea36e52d1027.shtml.

[10] Department of Science, Technology and Education (2014). Residency Training has a National Standard. http://www.nhc.gov.cn/qjjys/s3594/201409/f69d5617b7914c82a60514be769ee63f.shtml.

[11] Fang H., Wei L., Mao J., Jia H., Li P., Li Y., Fu Y., Zhao S., Liu H., Jiang K. (2020). Extent and risk factors of psychological violence towards physicians and Standardised Residency Training physicians: A Northern China experience. Health Qual. Life Outcomes.

[12] Zhang Y., Huang X., Li H., Zeng X., Shen T. (2019). Survey results of job status of residents in a standardized residency training program. BMC Med. Educ..

[13] Tang C., Tang D. (2018). The trend and features of physician workforce supply in China: After national medical licensing system reform. Hum. Resour. Health.

[14] Huynh A., Savitski J., Kirven M., Godwin J., Gil K.M. (2011). Effect of Medical Students’ Experiences with Residents as Teachers on Clerkship Assessment. J. Grad. Med. Educ..

[15] Frey-Vogel A. (2020). A Resident-as-Teacher Curriculum for Senior Residents Leading Morning Report: A Learner-Centered Approach Through Targeted Faculty Mentoring. MedEdPORTAL.

[16] Wu L., Qi H., Wu J., Li J. (2013). Analysis on different general practitioner training mode effect. Chin. J. Health Educ..

[17] Wang J., Song M., Gao P., Gu Z., Jia M., Yao S., Li N., Wang C. (2015). Survey of residency capability enhancement following standardized training. Chin. J. Hosp. Adm..

[18] Chen L., Wang L., Ni W. (2016). Satisfaction analysis and policy recommendations of standardized training of specialist doctors in Shanghai Jiaotong University School of Medicine. Contin. Med. Educ..

[19] Geary A., Hess D.T., Pernar L.I. (2019). Resident-as-teacher programs in general surgery residency—A review of published curricula. Am. J. Surg..

[20] Huang L., Hu S., Wang H., Cui H., Jin L. (2017). Study on the relation of job burnout and turnover intention in the resident physician of general standardized training program. Shanghai Med. Pharm. J..

[21] Musunuru S., Lewis B., Rikkers L.F., Chen H. (2007). Effective Surgical Residents Strongly Influence Medical Students to Pursue Surgical Careers. J. Am. Coll. Surg..

[22] Organ D.W., Near J.P. (1985). Cognition vs. affect in measures of job satisfaction. Int. J. Psychol..

[23] Smith P.C., Kendall L.M., Hulin C.L. (1969). The Measurement of Satisfaction in Work and Retirement.

[24] Zhang Z., Wu C., Zhang C., Zhong Y., Huang L. (2016). Investigation on the current situation and influencing factors of the satisfaction degree of resident physicians in standardized training in Shanghai. Med. Educ. Manag..

[25] Fan Y., Zhu Y., Zhang Q., Zhou X., Xie D., Xiong W., Zhang Y., Bao P. (2019). Satisfaction survey of students in standardized resident training in Guangdong Province. Res. Health Econ..

[26] Gu J., Zhao C., Luo X., Li D., Gu M. (2021). A survey of factors influencing the satisfaction of residents in a tertiary hospital in Chongqing on standardized training. J. Nanjing Med. Univ. (Soc. Sci. Ed.).

[27] Zhejiang Medical Ethics Network (2020). Zhejiang Province Clinical Trial Ethics Collaborative Review Alliance Consensus (First Edition). https://lunli.wsjkw.zj.gov.cn/interIndex.do?method=draftinfo&draftId=2c91d463-71c9d358-0171-c9d358b5-0000.

[28] Office for Human Research Protection (OHRP) 45 CFR 46. Office for Human Research Protections. https://www.hhs.gov/ohrp/regulations-and-policy/regulations/45-cfr-46/index.html.

[29] Kafetsios K., Anagnostopoulos F., Lempesis E., Valindra A. (2013). Doctors’ Emotion Regulation and Patient Satisfaction: A Social-Functional Perspective. Heal. Commun..

[30] Shen Y., Tan S., Wang B., Jin Y., Schläger C. (2014). Family Changes in China and Comparative Research of Family Policies.

[31] Han X. (2012). Income Inequalities within Couples in China. Online First Stat. Res..

[32] Qing S. (2019). Social-Cultural Roots of Gender Income Difference in China: Evidence from the Gender Role Attitudes. Online First Sociol. Stud..

[33] Nie L. (2012). Gender Differences in Work Values in China. Master’s Thesis.

[34] Wisener K.M., Driessen E.W., Cuncic C., Hesse C.L., Eva K.W. (2020). Incentives for clinical teachers: On why their complex influences should lead us to proceed with caution. Med. Educ..

[35] Esteghamati A., Baradaran H., Monajemi A., Khankeh H.R., Geranmayeh M. (2016). Core components of clinical education: A qualitative study with attending physicians and their residents. J. Adv. Med. Educ. Prof..

[36] Kelekar A., Afonso N. (2020). Evaluation of the effect of a new clinical reasoning curriculum in a pre-clerkship clinical skills course. Perspect. Med. Educ..

[37] Lio J., Ye Y., Dong H., Reddy S., McConville J., Sherer R. (2017). Standardized residency training in China: The new internal medicine curriculum. Perspect. Med. Educ..

[38] Science T., Division E. (2016). Notice of the Office of the National Health and Family Planning Commission of Issuing Standardized Residency Training Base Standards and Standardized Residency Training Content and Standards. http://www.nhfpc.gov.cn/qjjys/s3593/201408/946b17f463fa4e5dbcfb4f7c68834c41.shtml.

[39] Leonard R. (2010). Working Lives of Chinese Physicians. https://repository.upenn.edu/uhf_2010/11.

[40] Dornan T., Bentley S.R., Kelly M. (2020). Medical teachers’ discursive positioning of doctors in relation to patients. Med. Educ..

[41] Li X., Lin Z. (2013). Transformational, Transactional Leadership and Leadership Effectiveness: The Mediating Effects of Leader-member Exchange. J. Chongqing Univ. (Soc. Sci. Ed.).

[42] Zhang P., Tao H., Sun Y. (2017). A logical analysis of the reform of physicians’ compensation system in public hospitals in China: A case study of Sanming City, Fujian Province. Chin. J. Health Policy.

[43] Liu C., Ouyang W., Wu J. (2020). A Survey on the Satisfaction of Standardized Residency Training in Chongqing. Open J. Soc. Sci..

[44] Wu J., Li J., Wang G. (2014). Questionnaire survey and needs analysis of the professional degree education in residency standardized training. Chin. J. Med. Educ..

[45] Ark T.K., Brooks L.R., Eva K.W. (2006). Giving Learners the Best of Both Worlds: Do Clinical Teachers Need to Guard against Teaching Pattern Recognition to Novices?. Acad. Med..

