# Supplementary material for: Satisfaction of Clinical Teachers on Standardized Residency Training Program (SRTP) in China: A Cross-Sectional Survey
Source: Int J Environ Res Public Health. 2022 May 6;19(9):5676. doi: 10.3390/ijerph19095676 (PMC9101658; doi:10.3390/ijerph19095676)
Supplement: Supplementary file 1 [file ijerph-19-05676-s001.zip › ijerph-1660281-supplementary.pdf]

Supplementary File S1

Univariate analysis include all variables (complected version)

Items		Sex			Marital status		
		Male	Female		Married or cohabited	Others	
**The SRTP provides enough supported content (e.g., number of diseases and cases) to meet teaching requirements.**	TRUE	275	278	χ^2=0.811 p=0.057	539	14	p=1.000
	FALSE	11	10		21	0	
**The newly developed design of the SRTP is reasonable.**	TRUE	241	255	χ^2=2.234 p=0.135	486	10	p=0.109
	FALSE	45	33		74	4	
**There is no problem with personnel policy by the SRTP.**	TRUE	242	257	χ^2=2.697 p=0.101	485	14	p=0.234
	FALSE	44	31		75	0	
**The SRTP does not cause conflicts between residents' studies and work.**	TRUE	181	213	χ^2=7.020 p=0.008	388	6	p=0.044
	FALSE	105	75		172	8	
**Leaders of medical institutions attach great importance to SRTP.**	TRUE	222	255	χ^2=12.183 p=0.000	465	12	p=1.000
	FALSE	64	33		95	2	
**The SRTP helps improve residents' abilities.**	TRUE	271	272	χ^2=0.027 p=0.869	530	13	p=0.545
	FALSE	15	16		30	1	
**The required training period for residents is not too long in the SRTP.**	TRUE	145	157	χ^2=0.838 p=0.360	296	6	χ^2=0.548 p=0.459
	FALSE	141	131		264	8	
**The residents have high motivations to attend daily training.**	TRUE	167	182	χ^2=1.389 p=0.239	340	9	χ^2=0.073 p=0.787
	FALSE	119	106		220	5	
**The residents' wage is good enough provided by SRTP.**	TRUE	122	138	χ^2=1.602 p=0.206	253	7	χ^2=0.128 p=0.720
	FALSE	164	150		307	7	
**Clinical teachers' subsidies supported by SRTP are good.**	TRUE	216	246	χ^2=8.941 p=0.003	451	11	p=0.742
	FALSE	70	42		109	3	

Items		Age				Education Level		
		≤40	41~50	51~60		Junior college degree or college	Postgraduate or above	

**The SRTP provides enough supported content (e.g., number of diseases and cases) to meet teaching requirements.**	TRUE	284	226	43	χ^2=0.958 p=0.619	16	5	χ^2=0.000 p=0.995
	FALSE	13	7	1		421	132	
**The newly developed design of the SRTP is reasonable.**	TRUE	261	194	41	χ^2=4.23 p=0.121	376	120	χ^2=0.213 p=0.644
	FALSE	36	39	3		61	17	
**There is no problem with personnel policy by the SRTP.**	TRUE	256	204	39	χ^2=0.334 p=0.846	383	116	χ^2=0.811 p=0.368
	FALSE	41	29	5		54	21	
**The SRTP does not cause conflicts between residents' study and work.**	TRUE	203	161	30	χ^2=0.039 p=0.981	314	80	χ^2=8.778 p=0.003
	FALSE	94	72	14		123	57	
**Leaders of medical institutions attach great importance to SRTP.**	TRUE	253	190	34	χ^2=2.385 p=0.304	362	115	χ^2=0.091 p=0.763
	FALSE	44	43	10		75	22	
**The SRTP helps improve residents' abilities.**	TRUE	285	216	42	χ^2=2.777 p=0.249	413	130	χ^2=0.030 p=0.863
	FALSE	12	17	2		24	7	
**The required training period for residents is not too long in the SRTP.**	TRUE	169	111	22	χ^2=4.624 p=0.099	224	78	χ^2=1.348 p=0.246
	FALSE	128	122	22		213	59	
**The residents have high motivations to attend daily training.**	TRUE	187	136	26	χ^2=1.215 p=0.545	273	76	χ^2=2.143 p=0.143
	FALSE	110	97	18		164	61	
**The residents' wage is good enough provided by SRTP.**	TRUE	145	95	20	χ^2=3.414 p=0.181	202	58	χ^2=0.636 p=0.425
	FALSE	152	138	24		235	79	
**Clinical teachers' subsidies supported by SRTP are good.**	TRUE	246	183	11	χ^2=2.442 p=0.295	350	112	χ^2=0.183 p=0.669
	FALSE	51	50	33		87	25	

**Items**	**Hospital level**			**Type of hospital**		
	**Third-class**	**Other**		**General hospital**	**TCM hospital**	**Specialized hospital**

**The SRTP provides enough supported content (e.g., number of diseases and cases) to meet teaching requirements.**	TRUE	217	336	χ^2=1.459 p=0.227	370	106	77	χ^2=1.936 p=0.380
	FALSE	11	10		11	6	4	
**The newly developed design of the SRTP is reasonable.**	TRUE	192	304	χ^2=1.560 p=0.212	336	91	69	χ^2=3.670 p=0.160
	FALSE	36	42		45	12	21	
**There is no problem with personnel policy by the SRTP.**	TRUE	190	309	χ^2=4.317 p=0.038	339	87	73	χ^2=10.571 p=0.005
	FALSE	38	37		42	25	8	
**The SRTP does not cause conflict between residents' study and work.**	TRUE	152	242	χ^2=0.685 p=0.408	270	65	59	χ^2=7.392 p=0.025
	FALSE	76	104		111	47	22	
**Leaders of medical institutions attach great importance to SRTP.**	TRUE	178	299	χ^2=6.817 p=0.009	326	83	68	χ^2=8.139 p=0.017
	FALSE	50	47		77	29	13	
**The SRTP helps improve residents' abilities.**	TRUE	218	325	χ^2=0.762 p=0.383	359	107	77	χ^2=0.330 p=0.848
	FALSE	10	21		22	5	4	
**The required training period for residents is not too long in the SRTP.**	TRUE	106	196	χ^2=5.686 p=0.017	216	48	38	χ^2=7.875 p=0.019
	FALSE	122	150		165	64	43	
**The residents have high motivations to attend daily training.**	TRUE	129	220	χ^2=2.830 p=0.093	237	62	50	χ^2=1.737 p=0.420
	FALSE	99	126		144	50	31	
**The residents' wage is good enough provided by SRTP.**	TRUE	91	169	χ^2=4.425 p=0.035	187	41	32	χ^2=6.711 p=0.035
	FALSE	137	177		194	71	49	
**Clinical teachers' subsidies supported by SRTP are good.**	TRUE	182	280	χ^2=0.106 p=0.745	303	91	68	χ^2=0.884 p=0.643
	FALSE	46	66		78	13	21	

**Items**	**Professional Title**	**Teaching experience**

		Attending physician	Deputy chief physician	Chief physician		<=10 years	11-20 years	>20 years	
**The SRTP provides enough supported content (e.g., number of diseases and cases) to meet teaching requirements.**	TRUE	222	217	114	χ^2=0.068 p=0.967	435	94	22	χ^2=1.862 p=0.394
	FALSE	9	8	4		19	2	0	
**The newly developed design of the SRTP is reasonable.**	TRUE	204	197	95	χ^2=4.463 p=0.107	391	82	21	χ^2=1.639 p=0.441
	FALSE	45	28	23		63	14	1	
**There is no problem with personnel policy by the SRTP.**	TRUE	196	257	99	χ^2=4.602 p=0.100	394	84	19	χ^2=0.041 p=0.980
	FALSE	35	21	19		60	12	3	
**The SRTP does not cause conflicts between residents' study and work.**	TRUE	163	159	72	χ^2=4.012 p=0.135	307	71	14	χ^2=1.730 p=0.421
	FALSE	68	66	46		147	25	8	
**Leaders of medical institutions attach great importance to SRTP.**	TRUE	201	182	94	χ^2=4.296 p=0.117	377	83	15	χ^2=4.245 p=0.120
	FALSE	30	43	24		77	13	7	
**The SRTP helps improve residents' abilities.**	TRUE	221	214	108	χ^2=2.817 p=0.245	431	89	21	χ^2=0.800 p=0.670
	FALSE	10	11	10		23	7	1	
**The required training period for residents is not too long in the SRTP.**	TRUE	133	111	58	χ^2=3.819 p=0.148	235	51	14	χ^2=1.208 p=0.547
	FALSE	98	114	60		219	45	8	
**The residents have high motivations to attend daily training.**	TRUE	149	139	61	χ^2=5.523 p=0.063	282	50	15	χ^2=3.883 p=0.143
	FALSE	82	86	57		172	46	7	
	TRUE	121	97	42		209	40	10	χ^2=0.611

**The residents' wage is good enough provided by SRTP.**	FALSE	110	128	76	χ^2=9.596 p=0.008	245	56	12	p=0.737
**Clinical teachers' subsidies supported by SRTP are good.**	TRUE	189	181	92	χ^2=0.738 p=0.691	363	80	17	χ^2=0.718 p=0.698
	FALSE	42	44	26		91	16	5	

**Items**		**Department**			
		**Internal medicine**	**Surgical**	**Others**	
**The SRTP provides enough supported content (e.g., number of diseases and cases) to meet teaching requirements.**	TRUE	64	192	297	χ^2=5.411 p=0.067
	FALSE	3	12	6	
**The newly developed design of the SRTP is reasonable.**	TRUE	53	176	267	χ^2=3.802 p=0.149
	FALSE	14	28	36	
**There is no problem with personnel policy by the SRTP.**	TRUE	56	176	267	χ^2=1.115 p=0.573
	FALSE	11	28	36	
**The SRTP does not cause conflicts between residents' study and work.**	TRUE	39	135	220	χ^2=6.177 p=0.046
	FALSE	28	69	83	
**Leaders of medical institutions attach great importance to SRTP.**	TRUE	50	170	257	χ^2=4.070 p=0.131
	FALSE	17	34	46	
**The SRTP helps improve residents' abilities.**	TRUE	64	193	286	χ^2=0.138 p=0.933
	FALSE	3	11	17	
**The required training period for residents is not too long in the SRTP.**	TRUE	32	105	165	χ^2=1.152 p=0.562
	FALSE	35	99	138	
**The residents have high motivations to attend daily training.**	TRUE	28	129	192	χ^2=11.503 p=0.003
	FALSE	39	75	111	
**The residents' wage is good enough provided by SRTP.**	TRUE	30	83	147	χ^2=3.024 p=0.220
	FALSE	37	121	156	
**Clinical teachers' subsidies supported by SRTP are good.**	TRUE	47	167	248	χ^2=5.163 p=0.076
	FALSE	20	37	55	

Declaration of IRB Exemption

Be guided by the principles contained in The Code of Federal Regulations governing research with human subjects (45 CFR 46)[1] and Operational Guidelines for Zhejiang Ethics Committees That Review Biomedical Research

(Version I)[2], all of authors agreed to register the study "*Satisfaction of clinical teachers on Standardized Residency Training Program in China: a cross-sectional survey*" as an institutional ethics exemption:

> "Research involving the use of educational tests (cognitive, diagnostic, aptitude, or achievement tests), survey procedures, interview procedures or observation of public behavior if:
>
> > *The information is gathered in such a manner that subjects cannot be identified, either directly (such as if you use photographs, video tapes, or voice recordings) or indirectly through identifiers (e.g., codes) linked to individuals; and*
> >
> > *Any disclosure of the subjects' responses outside of the research will not be damaging to the subject in any way (i.e., subject him/her to criminal or civil liability, damage financial standing, reputation, etc)".*[1]
>
> "Research conducted in established or commonly accepted educational settings, involving normal educational practices, such as 1) research on instructional strategies; 2) research on the effectiveness of or the comparison among instructional techniques, curricula, or classroom management methods.".[2]

Furthermore, all of authors promise the study's risks are minimal and that subjects' identities are protected. All members of the research team completed the required online training module through Collaborative Institutional Training Initiative (CITI Program). The detailed research information described in the below sheet:

**Research Summary**	
Purpose:	The purpose of this study is to analyze critical factors affecting clinical teachers' satisfaction and provide continuous improvements for standardized residency training program (SRTP).
Methodology:	The objective of the study is to better understand clinical teachers' cognitive job satisfactions. In order to do so we administered a self- designed survey to clinical teachers of 13 residency standardization bases in Shaoxing City, Zhejiang Province, China. The survey took approximately 20 minutes to complete. Participants had to have teaching experience of over one year. There is an informed content attached includes

	the brief introduction of study's purpose and measurements. We received the information and/or record the information in such a way that subjects cannot be identified. The self-administered questionnaire had 19 items, respectively, which included three sections:<br>(1) Demographic information (gender, age, marital status (*recoded into married and other [including unmarried, divorced, and widowed]*)), and education level (*junior college or college, and postgraduate or above*);<br>(2) Career information (working experience (*10-year categories*), professional titles (*attending physician, deputy chief physician, and chief physician*), hospital types (*TCM hospital, specialized hospital, and general hospital*), hospital levels (*tertiary hospital, and non-tertiary hospital*), and clinical departments (*department of internal medicine, surgical department, and others*), and<br>(3) Perceived job satisfaction (*evaluated the satisfaction into positive attitude (PA) and negative attitude (NA)*).
Study Population:	Our study population included clinical teachers of 13 residency training bases we had permissions to recruit from. The inclusion certification required all recruited participants to have teaching experience of over one year. Both males and females will be included and no one will be excluded based on ethnicity or race, education status or socioeconomic status.
Research Specific Risks:	There were no physical risks associated with this study, but there was a possible risk of loss of confidentiality or privacy of the subjects. However, since this was an anonymous survey, no identifiable information will be collected. The specific procedures for maintaining confidentiality and privacy are outlined below in the section "Confidentiality and privacy for the subjects".
Uses of data:	Only the researchers accessed to the data, and since no identifying information was collected in this study, there would not be any identifying information used in any publications. At the completion of the study, the surveys collected would be disposed of by shredding them.
Subject Recruitment:	We used a recruitment script* (attached under the sheet), that shared and presented to clinical teachers online. It gave a brief description of the study including purpose, time

	commitment and any risks. They also received more detailed information of the study.
Justification of Sample Size:	The target number of participants we needed in order to answer my research question is 600, however, since the survey conducted online more or less people may participate depending on the response rate and interest. The statistical method used to analyze the data was SPSS18.0 software.
Confidentiality and privacy for the subjects:	The data will be collected in such manner that subjects cannot be identified. The only information I intend on extracting from the records is the subject's sex, age, marriage status and education level. This information is not enough to identify the research subject. There are no codes in existence which will allow the researcher to link the data back to the subject.

***Informed Content:**

"Greetings to all the instructors! It has been seven years since the implementation of the standardized residency training program (SRTP) in our country. As a clinical teacher, you must have an unique perspective to understand the current situations and issues from the SRTP. The purpose of this survey is to evaluate your job cognitive satisfaction of SRTP, and to provide a basis for further development on Chinese standardized residency training. Please fill out the questions in the survey independently, according to your knowledge and personal wishes.

The research team promises to keep the contents of the survey confidential, and all of questionnaires were answered anonymous. Except for the number of optional items marked, the multiple- choice questions in the table are all single choice (please mark "√" on the serial number of the item selected).

Thank you very much for your support to our work!"
